# Parameters in Dynamic Models of Complex Traits are Containers of Missing Heritability

**DOI:** 10.1371/journal.pcbi.1002459

**Published:** 2012-04-05

**Authors:** Yunpeng Wang, Arne B. Gjuvsland, Jon Olav Vik, Nicolas P. Smith, Peter J. Hunter, Stig W. Omholt

**Affiliations:** 1 Centre for Integrative Genetics, Department of Animal and Aquacultural Sciences, Norwegian University of Life Sciences, Ås, Norway; 2 Centre for Integrative Genetics, Department of Mathematical Sciences and Technology, Norwegian University of Life Sciences, Ås, Norway; 3 Department of Biomedical Engineering, St Thomas' Hospital, King's College London, London, United Kingdom; 4 Auckland Bioengineering Institute, The University of Auckland, Auckland, New Zealand; Harvard Medical School, United States of America

## Abstract

Polymorphisms identified in genome-wide association studies of human traits rarely explain more than a small proportion of the heritable variation, and improving this situation within the current paradigm appears daunting. Given a well-validated dynamic model of a complex physiological trait, a substantial part of the underlying genetic variation must manifest as variation in model parameters. These parameters are themselves phenotypic traits. By linking whole-cell phenotypic variation to genetic variation in a computational model of a single heart cell, incorporating genotype-to-parameter maps, we show that genome-wide association studies on parameters reveal much more genetic variation than when using higher-level cellular phenotypes. The results suggest that letting such studies be guided by computational physiology may facilitate a causal understanding of the genotype-to-phenotype map of complex traits, with strong implications for the development of phenomics technology.

## Author Summary

Despite an ever-increasing number of genome locations reported to be associated with complex human diseases or quantitative traits, only a small proportion of phenotypic variations in a typical quantitative trait can be explained by the discovered variants. We argue that this problem can partly be resolved by combining the statistical methods of quantitative genetics with computational biology. We demonstrate this for the *in silico* genotype-to-phenotype map of a model heart cell in conjunction with publically accessible genomic data. We show that genome wide association studies (GWAS) on model parameters identify more causal variants and can build better prediction models for the higher-level phenotypes than by performing GWAS on the higher-level phenotypes themselves. Since model parameters are in principle measurable physiological phenotypes, our findings suggest that development of future phenotyping technologies could be guided by mathematical models of the biological systems being targeted.

## Introduction

The phenotypic variance cumulatively explained by marker loci found to associate with complex traits in genome-wide association studies (GWAS) is usually much less than the narrow-sense heritability [1]–[6], the ratio of additive genetic variance to total phenotypic variance. Several explanations have been proposed for this unexplained variance, popularly called the missing heritability [1], including imprecise heritability estimates; insufficient sample size; exclusion of particular types of polymorphisms such as copy number variants and rare SNPs in GWAS; unaccounted epistatic effects; and underestimated effect size of associated SNPs due to incomplete linkage with causal variants [3], [6]. Recently it was shown [7] that a large proportion of the missing heritability can be accounted for if one estimates the variance explained by all available marker loci together. This suggests that most of the genetic variation underlying complex trait variation is due to marginal effects of many loci that are too small to pass stringent significance tests. Strong support for this interpretation comes from several meta-analyses of genome-wide association data [8]–[10]. While this insight appears to resolve much of the missing heritability issue as such, it also implies that standard GWAS approaches will not be very helpful for disclosing which genetic variants do actually contribute to additive variance.

Part of the problem underlying the missing heritability is that while the genotype-phenotype map in reality arises from complex biological systems best described by nonlinear dynamic models, the statistical machinery of quantitative genetics, including GWAS methods, is built upon linear models of gene action. The aim of this study is not to improve the statistical methods *per se*, but rather to explore how more of the missing heritability can be explained and understood by combining nonlinear dynamic models with existing GWAS methods. The research program of linking system dynamics and genetics was suggested more than 40 years ago [11] and has been an active research area for more than 10 years [12]–[24]. Emergent properties of nonlinear systems, such as systemic silencing [25], might lead to a situation where genetic variation that penetrates to low-level phenotypes underlying a higher-level phenotype does not necessarily manifest in the higher-level phenotype itself. Doing GWAS on these low-phenotypes may thus reveal more of the genetic variation influencing the higher-level trait. This line of reasoning is reflected in recent GWA studies on metabolite profiles [26], [27], in pathway and network-based analysis of genome-wide association studies [28] and in GWAS analyses on global gene expression data [29]–[30]. While all these studies represent important contributions, they do not combine a genetic mapping framework with mathematical models describing how high-level trait variation emerges from low-level trait variation, i.e. they do not provide a quantitative framework for elucidating how genetic variation affecting a low-level phenotype do actually influence a focal high-level phenotype.

If a dynamic model can describe the phenotypic variation of a given trait, it follows that irrespective of the biological resolution of the model, the genetic variation underlying the phenotypic variation will have to be reflected as variation in the parameters of the model. We therefore hypothesized that performing GWAS on parameters in computational physiology models might reveal much more of the underlying genetic variation, as well as shedding light on how this variation actually causes phenotypic variation.

To test the plausibility of this reasoning, we combined GWAS methodology with a causally-cohesive genotype-phenotype (cGP) model linking genetic variation to phenotypic variation. More specifically, a cGP model [19] is a mathematical model of a biological system where low-level parameters have an articulated relationship to an individual's genotype, and higher-level phenotypes emerge from the mathematical model describing the causal dynamic relationships between these lower-level processes. Our approach bears some resemblance to that of functional GWAS (fGWAS) [31], where the genetic control of traits is analyzed by integrating biological principles of trait formation into the GWAS framework through mathematical and statistical bridges. Fu *et al.* [23] recently extended the functional mapping framework [15] to handle cyclic phenotypes such as circadian rhythms by combining differential equations with functional mapping of QTLs. However, there are clear differences between functional mapping and the cGP approach. In functional mapping the phenotypic measurements are currently done at the systems level, while lower-level parameters are estimated by combining curve-fitting with more classical QTL methods. In contrast, the cGP approach advocated here focuses on measuring lower-level parameters based on the idea that they are highly relevant phenotypes of the system.

**Figure 1:**
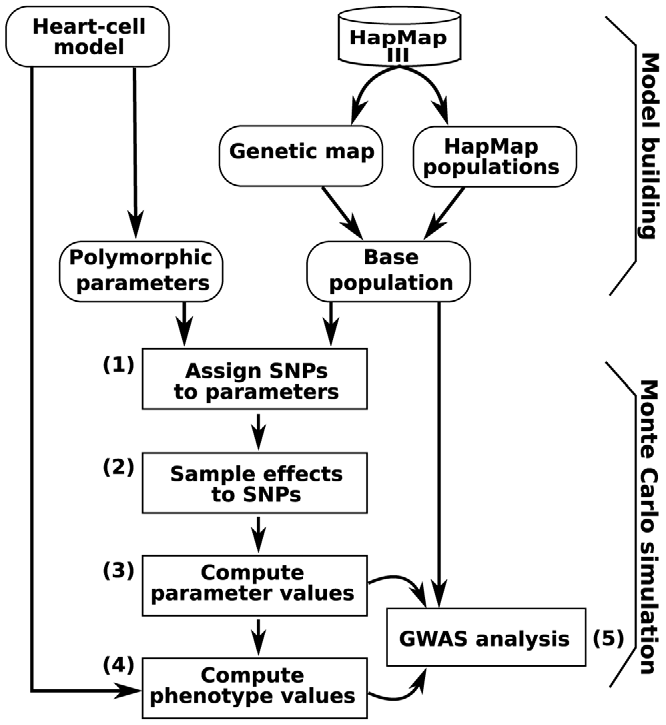
Flowchart of computational pipeline. A heart cell model, a genetic map and a virtual population are tied together by selecting heart model parameters assumed to be under influence of genetic variation and associating the parameter variation to a population of virtual genomes based upon HapMap 3 data. Individual genotypes are mapped into heart model parameters (steps 1–3) and by running the heart cell model parameters are mapped into cell-level phenotypes (step 4). Finally, GWAS analysis is then performed on the virtual population (step 5).

We studied a cGP model of a mouse heart cell [24], where genetic variation is mapped to parametric variation, which propagates through the physiological model to generate multivariate phenotypes for the action potential (an electrical signal) and calcium transient (linked to muscle contraction) under regular pacing. The rationale for using a heart cell model was that multiscale and multiphysics modelling of the mammalian heart has a solid empirical basis, and arguably comprises the most complex mathematical conceptualization of any organ or physiological trait available. For clarity of exposition, and because heart cell models lie at the core of this class of multiscale whole organ models [32]–[37], we deemed it sufficient to illustrate our points using a single cell model only. We used HapMap data [38], [39] as a guide to generate genetic variation with realistic allele frequencies and linkage disequilibrium to underlie variation in the model parameters. Based on HapMap [39] individuals we simulated complex pedigree populations and performed GWAS on both low-level parameters and high-level phenotypes arising from the cGP model. The layout of the computational pipeline used for this study is depicted in Figure 1.

We show that genome-wide association studies on parameters reveal many more of the underlying SNPs than when using higher-level cellular phenotypes. Furthermore, the SNPs identified by GWAS on parameters can be used to build multivariate prediction models of higher-level phenotypes giving much higher explained variance than from GWAS on higher-level phenotypes alone. Our results suggest that combining statistical genetics with computational biology will facilitate both identification of genetic variation underlying complex traits and a much deeper understanding of how this genetic variation becomes causative.

## Methods

The general layout of the study is outlined in Figure 1.

### Heart cell model

The cell model [40] extends that of Bondarenko *et al.* [41] with more realistic calcium handling, conservation of charge, and detailed re-parameterization to consistent experimental data for the C57BL/6 “black 6” mouse. State variables include ion concentrations of sodium, potassium and calcium in the cytosol, calcium concentration in the sarcoplasmic reticulum, and the state distribution of ion channels, whose transition rates between open, closed, and inactivated conformations may depend on transmembrane voltage. Formulated as a system of coupled ordinary differential equations, this model provides a comprehensive representation of membrane-bound channels and transporter functions as well as fluxes between the cytosol and intracellular organelles. As the action potential and calcium transient features following an electrical stimulation are the only state descriptors fed into higher level features of current multiscale heart models [33], we used these and associated aggregated measures as high-level phenotypes, see “Parameter to phenotype mapping” below. See Vik *et al.* [24] for a detailed description of this model including model diagram, differential equations and a CellML implementation.

### Polymorphic parameters

Out of the 86 model parameters we chose 34 to mediate the effects of genetic variation (Table 1 and Table S1). Because the genotype to parameter map for parameters describing ion channel properties may in general be much more straightforward than what is the case for many others, we picked mainly parameters describing affinities, conductivities and ion permeabilities for the ion channels and pumps underlying four potassium outward currents, one calcium current, one chloride current, one sodium current, the sodium-calcium exchangers, the sarcoplasmic reticular calcium ATPase (SERCA), the sodium potassium pump, cytosolic calmodulin, the ryanodine receptors on sarcoplasmic reticulum and the calcium handling processes within sarcoplasmic reticulum.

**Table 1:** Parameters with genetic variation.

Parameter name	Description	Unit	Baseline value	Min	Max
**Ka+**	The PC1 – PO1 rate constant of the Ryanodine receptor	µM^−4^/ms	6.08e-3	4.09e-3	8.06e-3
**Ka−**	The PO1 – PC1 rate constant of the Ryanodine receptor	ms^−1^	7.133-2	4.70e-2	9.58e-2
**Kb+**	The PO1 – PO2 rate constant of the Ryanodine receptor	µM^−3^/ms	4.05e-3	2.64e-3	5.47e-3
**Kb−**	The PO2 – PO1 rate constant of the Ryanodine receptor	ms^−1^	9.65e-1	6.32e-1	1.31
**Kc+**	The PO1 – PC2 rate constant of the Ryanodine receptor	ms^−1^	9.00e-3	6.09e-3	1.20e-2
**Kc−**	The PC2 – PO1 rate constant of the Ryanodine receptor	ms^−1^	8.00e-4	5.24e-4	1.07e-3
**m**	The Ca^2+^ cooperativity parameter of PO1 – PO2 of the Ryanodine receptor	-	3.0	1.99	3.97
**n**	The Ca^2+^ cooperativity parameter of PC1 – PO1 of the Ryanodine receptor	-	4.0	2.75	5.33
**P_CaL**	The permeability of the L-type Ca^2+^ channel	ms^−1^	2.5	1.62	3.30
**t_L**	The time constant of the switch between open and close states of the L-type Ca^2+^ channel	ms^−1^	1.5	1.01	1.98
**tau_L**	The Inactivation time constant of the L-type Ca^2+^ channel	ms^−1^	1.15e3	7.82e2	1.52e3
**phi_L**	The proportion of closed states in open mode of the L-type Ca^2+^ channel	-	1.80	1.23	2.43
**Kup**	The SERCA affinity to Ca^2+^	µM	4.12e-1	2.93e-1	5.68e-1
**V1**	The leak constant of the Network Sarcoplasmic Reticulum	ms^−1^	4.5	3.05	5.90
**KCSQN**	The Calsequestrin affinity to Ca^2+^	µM	6.30e2	4.35e2	8.57e2
**K_Co**	The affinities of the Na^+^/Ca^2+^ exchanger to extracellular Ca^2+^	µM	1.4e3	9.38e2	1.85e3
**K_Ci**	The affinities of the Na^+^/Ca^2+^ exchanger to intracellular Ca^2+^	µM	3.6	2.45	4.93
**K_No**	The affinities of the Na^+^/Ca^2+^ exchanger to extracellular Na^+^	µM	8.80e4	6.06e4	1.20e5
**K_Ni**	The affinities of the Na^+^/Ca^2+^ exchanger to intracellular Na^+^	µM	1.2e4	8.38e3	1.58e4
**KNai**	The affinity of the Na^+^/K^+^ pump to intracellular Na^+^	µM	1.66e4	1.13e4	2.17e4
**KKo**	The affinity of the Na^+^/K^+^ pump to extracellular K^+^	µM	1.5e3	1.04e3	2.08e3
**KpCa**	The affinity of the Ca^2+^ pump to intracellular Ca^2+^	µM	2.89e-1	1.95e-1	3.93e-1
**Vmax**	The maximal exchange rate of Na^+^/Ca^2+^ exchanger	pA/pF	3.94	2.71	5.19
**Imax**	The maximal current of the Na^+^/K^+^ pump	pA/pF	2.49	1.71	3.58
**GK1**	The maximal conductance of the time-dependent K^+^ channel	ms/µF	3.5e-1	2.39e-1	4.52e-1
**GKr**	The maximal conductance of the rapid delayed rectifier K^+^ channel	ms/µF	1.65e-2	1.11e-2	2.17e-2
**GKur**	The maximal conductance of the ultrarapidly activating delayed rectifier K^+^ channel	ms/µF	2.50e-1	1.76e-1	3.27e-1
**KCl**	The half saturation constant of the Ca^2+^ activated Cl^−^ channel	µM	1.00e1	6.65	1.36e1
**GNa**	The maximal conductance of the Na^+^ channel	ms/µF	1.60e1	1.07e1	2.10e1
**GKtof**	The maximal conductance of the rapidly recovering transient outward K^+^ channel	ms/µF	5.35e-1	3.97e-1	7.11e-1
**GClCa**	The maximum conductance of the Ca^2+^ activated Cl^−^ channel	ms/µF	1.00e1	6.56	1.33e1
**on_rate**	The autophosphorylation rate of Calmodulin	ms^−1^	5.0e-2	3.25e-2	6.56e-2
**off_rate**	The dephosphorylation rate of the Calmodulin	ms^−1^	2.0e-4	1.34e-4	2.67e-4
**IpCm**	The maximal current of the Ca^2+^ pump	pA/pF	9.55e-2	6.35e-2	1.26e-1

Listing of the 34 parameters where genetic variation was introduced. The descriptions, units and baseline values are taken from the original publication [40]. The minimum and maximum values were obtained from the Monte Carlo simulations.

### Virtual genome and virtual population

To ensure some realism in the construction of the genetic structure of our *in silico* populations in terms of allele frequencies and LD patterns, we extracted HapMap3 data [39] for 2,000 evenly spaced SNPs (∼5000 nucleotides apart) for each of the first 20 autosomal chromosomes. We extracted genotypes for the 40000 SNPs for the 1301 individuals in the 11 HapMap 3 populations. We then expanded this into a population of 5000 individuals by use of the Python package simuPOP [42]. The population expansion by simuPOP maintained allele frequencies and LD patterns in accordance with the HapMap 3 data. Mutations were introduced based on a symmetric diallelic mutation model, all recombinations were based on genetic maps estimated from the HapMap data and migrations between the 11 subpopulations were allowed for. The mutation rate, migration rate and number of generations used as input to the simuPOP population expansion were 1e-8, 0.001 and 500, respectively.

### Genotype to parameter mapping

Assuming a purely additive genetic model, 400 causal SNPs were randomly sampled from the virtual genome for each of the 34 parameters selected to mediate genetic variation. The genotype to parameter mapping for each parameter was set up by defining the 5,000×40,000 genotype matrix ***G***, where each element *g_ij_* denoted the genotype of individual *i* at SNP *j* (−1 for the homozygous with the least frequent allele, 0 for the heterozygous and 1 for the homozygous with the most frequent allele). We then constructed for each parameter the 40,000x1 relative effect vector ***E***, where element *e_j_* was sampled from a *Laplace* (0, 0.0035) distribution if the *j*-th SNP was among the 400 parameter-specific causative SNPs, and set to 0 otherwise (the relative effect being defined as the percentage increase or decrease of the baseline parameter value (Table 1 and Table S1)). The 5000-element vector of parameter values for all individuals was then computed as *p(* ***GE*** *+1)*, where *p* is the baseline value. With this procedure, each of the focal 34 parameters was varied within ∼±35% of its baseline value, and for each causal SNP, the heterozygous individuals were assigned the baseline parameter value (Table 1 and Table S1). The ±35% parameter variation range was chosen as a compromise between getting ample genetic signals and avoiding too many physiologically unrealistic phenotypes. We also tested a genetic model with 200 causative SNPs for each parameter, the only difference being that the standard deviation of the *Laplace* distribution was set to 0.0049.

### Parameter to phenotype mapping

Cellular phenotypes for individual parameter sets were generated by a virtual experiment of constant pacing as described in Bondarenko *et al.* [41]. The potassium current was stimulated by −15 V/s for 3 ms at the start of each stimulus interval. Convergence was checked by comparing successive intervals with respect to the initial value of each state variable as well as the integral of its trajectory over that interval. A running history of 10 intervals was kept, and after each interval we checked for a match (within a relative tolerance of 5% for all state variables) against the previous one. This was done for three different pacing rates with stimulus intervals of intervals 100, 200 and 300 ms, respectively. The cell dynamics was categorized as “failure” if it did not converge to non-alternating dynamics within 10 minutes of simulation time. The Python code of the heart cell model was autogenerated from CellML [43], using the code generating service available at the CellML repository (www.cellml.org). The equations were integrated using the CVODE solver [44] with a Python wrapper.

Eight scalar phenotypes (see Table 2 and Table S2) were extracted from each computed action potential and calcium transient curve: the initial value (apbase and ctbase), the amplitude (apamp and ctamp), the peak value (appeak and ctpeak), the time to peak value (apttp and ctttp), the time to 25%, 50%, 75%, and 90% of the initial base value (apd25, apd50, apd75, apd90 and ctd25, ctd50, ctd75, ctd90).

**Table 2:** Attained cellular phenotype values.

Phenotypes	Unit	Baseline value	Min	Max
**apd25**	ms	4.34	4.10	4.56
**apd50**	ms	5.89	5.33	6.39
**apd75**	ms	1.11e1	9.28	1.29e1
**apd90**	ms	1.95e1	1.60e1	2.30e1
**apamp**	mV	1.18e2	1.14e2	1.23e2
**apbase**	mV	−8.00e1	−8.0.6e1	−7.93e1
**appeak**	mV	3.82e1	3.41e1	4.23e1
**apttp**	ms	3.20	3.03	3.35
**ctd25**	ms	6.19e1	4.80e1	7.98e1
**ctd50**	ms	1.05e2	7.98e1	1.37e2
**ctd75**	ms	1.79e2	1.39e2	2.16e2
**ctd90**	ms	2.55e2	2.20e2	2.79e2
**ctamp**	µM	1.4e-1	4.85e-2	2.76e-1
**ctbase**	µM	8.14e-2	6.12e-2	1.05e-1
**ctpeak**	µM	0.22	1.15e-1	3.68e-1
**ctttp**	ms	2.40e1	1.93e1	2.98e1

The phenotypic values resulting from use of the baseline parameter values (see Table 1) are listed together with the minimum and maximum values achieved in the Monte Carlo simulations.

### Data preparation

We removed individuals with physiologically unrealistic phenotypes within each of the 100 datasets analyzed. The exclusion criterion was based on the inter-quartile range (IQR); points that were more than twice the IQR above the third quartile or below the first quartile were excluded. Each filtered data set, containing 4000–5000 individuals, was divided into a training set of 2500 individuals and a test set consisting of the remaining individuals. The training data set was used to detect causal SNPs, compute the false positive rate and sensitivity characteristics. The test set was used to estimate the phenotypic variation accounted for by the detected SNPs.

### Statistical analysis

The same GWAS procedure was used for each parameter and each phenotype. The quantitative trait association analysis was performed with the program PLINK [45] on the training data. We used a threshold of 1e-5 on the Bonferroni-corrected p-value from PLINK to determine the set of significant SNPs.

The detected SNP set and associated discovery rates were defined as follows. Let ***S*** *_i_* denote the set of significant SNPs from GWAS on the *i*-th parameter and let ***C*** *_i_* denote the causal SNPs set of the *i*-th parameter. The set of detected SNPs of the *i*-th parameter was then computed as ***D*** *_i_* = ***S*** *_i_*∩***C*** *_i_*, and the discovery rate of *i*-th parameter was computed as *d_i_* = |***D*** *_i_*|/|***C*** *_i_*|. The union of causal SNP sets for parameters defined the causal SNP set underlying all cellular phenotypes, and the detected SNP set and the discovery rate for each cellular phenotype was computed in the same way as for each parameter. The set of false positive SNPs of the *i*-th parameter or phenotype, ***F*** *_i_*, consists of SNPs in the set of significant SNPs ***S_i_*** that are not in the causal SNPs set ***C*** *_i_*_._. The false positive rate of the *i*-th parameter or phenotype was defined as the number of false positive SNPs in ***F*** *_i_* divided by the number of signals in ***S_i_***, |***F*** *_i_*|/|***S_i_***|.

To quantify explained genetic variance a multiple regression model was constructed by regressing the phenotype or parameter value of the training set on the causal SNPs detected by GWAS (similar to the weighted genomic profile approach in [46]). Then the phenotypes of test set individuals were predicted using the corresponding fitted models. We measured the explained variation by the R^2^ values from regressing observed values on predicted phenotypic values for the individuals in the test set.

### Global sensitivity analysis

We quantified the linear sensitivity [47] of each phenotype to each parameter using linear regression in the training set. For each high-level phenotype and Monte Carlo simulation we used the 2500 simulated phenotypes as response and performed a series of univariate regressions each time with a single parameter as predictor. We measured global sensitivity by the coefficient of determination (R^2^).

## Results/Discussion

**Figure 2:**
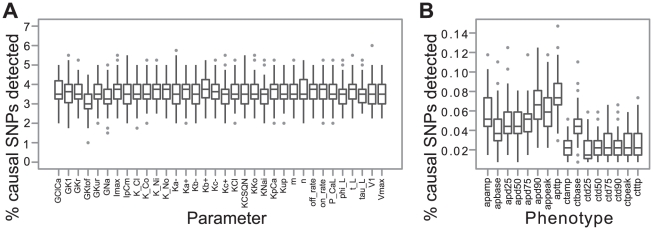
Percentage of causative SNPs detected by GWAS. (**A**) The percentage of 400 causative SNPs (y axis) detected as significant SNPs by GWAS on genetically controlled model parameters (x axis). (**B**) The percentage of all 13600 causative SNPs (y axis) detected as significant SNPs by GWAS on cellular phenotypes (x axis). Each boxplot summarizes 100 Monte Carlo runs. See Methods for further descriptions of model parameters and phenotypes.

The proportion of true causative SNPs detected by GWAS was as expected substantially higher for the parameters than for the cellular phenotypes (Figure 2 and Figure S2 for the 200 SNPs case). Median detection rates of causal SNPs were in the range 3.5%–4% after GWAS directly on parameter values (Figure 2A), and this number dropped to ∼0.05% for GWAS studies on action potential phenotypes and ∼0.02% for calcium transient phenotypes (Figure 2B), and the corresponding figures in the 200 SNPs case were 8–8.5%, ∼0.16% and ∼0.08%. The low overall detection rates were to be expected since we sampled SNP effects from an L-shaped distribution resulting in datasets where a small proportion of the SNPs underlying a given parameter will explain a substantial part of the variation. The main explanation for the decrease in detection rates is that the number of causal SNPs increases 34 times and the relative effects of all causal SNPs decrease, making them harder to pick up. Another, probably less important, phenomenon contributing to lower detection rates at the higher-level phenotypes is that going from parameter level to the system-level phenotype introduces nonlinearities in the SNP effects, and standard GWAS methods pick up only the additive part.

**Figure 3:**
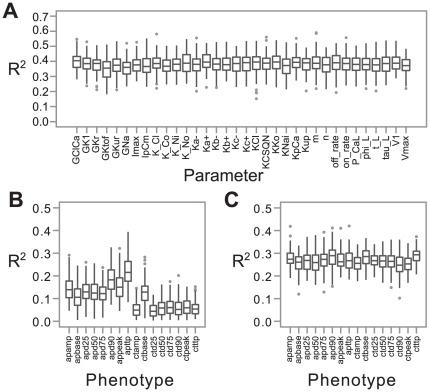
Phenotypic variance explained by genotypic variation. (**A**) Total explained variance for genetically controlled parameters (x axis) using detected causal SNPs as predictors. (**B**) Total explained variance for cellular phenotypes (x axis) using detected causal SNPs obtained from GWAS targeting these phenotypes. (**C**) Total explained variance for cellular phenotypes (x axis) using detected causal SNPs obtained from GWAS targeting all genetically controlled parameters. Each boxplot summarizes total explained variance by GWAS for 100 Monte Carlo runs. Explained variance was measured as R^2^ from test set prediction with a multiple regression model, see Methods for further descriptions.

The difference between parameter and cellular phenotypes is also evident when looking at the amount of phenotypic variance explained by SNPs detected in the GWAS (Figure 3 and Figure S3 for the 200 SNPs case). The median explained variance is typically in the range 30–40% for parameter phenotypes (Figure 3A), 10–20% for action potential phenotypes and ∼5% for calcium transient phenotypes (Figure 3B). The proportion of phenotypic variance explained by detected SNPs was on average 2.6 (2.0 in the 200 SNP case) and 5.6 (3.9 for the 200 SNPs case) times higher for a parameter phenotype than for an action potential and calcium transient phenotype, respectively. However, when we made use of the SNPs detected for parameters we were able to explain 1.8 and 3.9 times (1.6 and 2.9 times for the 200 SNPs case) more of the phenotypic variance of the action potential and calcium transient phenotypes, respectively, approaching the levels obtained for the parameters (Figure 3C). We also calculated the explained variances with all significant SNPs and obtained similar results. This suggests that our approach can be tested empirically in a straightforward way.

The gain in explained variance by using parameter-associated SNPs was not as dramatic for the action potential phenotypes as for the calcium transient phenotypes (Figure 3C), but even in this case the gain in number of identified SNPs was on average 13.9× (12.3 for the 200 SNPs case). The corresponding figure for the calcium transient phenotypes was 39.4× (26.5 for the 200 SNPs case). Because these additional SNPs are attached to one or more parameters describing specific biological processes or features that are causally related according to the functional structure of the mathematical model, the gain in our causal understanding of the genotype to phenotype map may be substantial.

**Figure 4:**
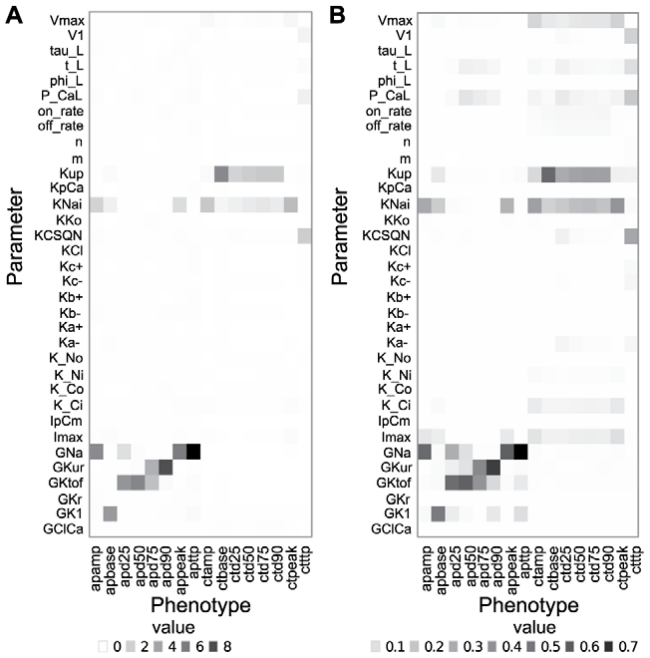
The close resemblance between GWAS results and linear sensitivity analysis. (**A**) The number of causative SNPs for each parameter(y axis) detected by performing GWAS on high-level cellular phenotypes(x axis). The color intensity of each square describes the mean value of 100 Monte Carlo runs. (**B**) Sensitivities of the high-level phenotypes (x axis) of the 2500 individuals in the training set to variation in each parameter (y axis) quantified by univariate linear regression (see Methods). The color intensity of each square describes the mean R^2^ (coefficient of determination) value of 100 Monte Carlo runs.

Both the detection rate of causal SNP and the variances explained for the calcium transient phenotypes were overall significantly lower than those for the action potential phenotypes (Figure 2B and 3B). We investigated this further by a linear global sensitivity analysis of how variation in the cellular phenotypes depended on variation in the parameters, and compared this with the number of causative SNPs for each parameter detected by performing GWAS on high-level cellular phenotypes. We found that the GWAS results for the two cellular phenotype groups are predominantly a consequence of the sensitivity structure of the dynamic model (Figure 4 and Figure S4 for the 200 SNPs case), and that the action potential phenotypes are overall more sensitive to fewer parameters than the calcium transient phenotypes. The only exception to this latter pattern is the parameter Kup, quantifying the affinity of SERCA to calcium ions (Figure 4A). It has a substantial impact on the calcium transient base value phenotype (ctbase), and the amount of variance explained by the SNPs detected for this phenotype is on par with the action potential phenotypes (Figure 3B). This suggests that SNPs associated with traits that are sensitive to few parameters will have a higher penetrance than SNPs associated with traits that are sensitive to many parameters for a given model resolution. Moreover, the results imply that the more poly-parametric the sensitivity profile of a model phenotype is, the more will be gained in terms of added explained variance by performing GWAS on parameters. On the other hand, the results also imply that a sensitivity analysis can be used to systematically reveal hotspots for genetic variation underlying a complex trait and thus guide a parameter phenotyping program. Within this framework a SNP affecting a parameter to which the focal higher-level phenotypes are not very sensitive will have little impact on these phenotypes unless it is highly penetrant at the parameter level.

GWAS methods are well known for producing false positives due to multiple testing and high LD between SNPs. A typical GWAS block of SNPs in high LD is often reduced to a subset of tagSNPs in low LD (typically with a pairwise correlation <0.2). The GWAS methods are aimed at identifying significant tagSNPs, and the task of distinguishing the causal SNPs from false positives in high LD has to be done with other methods such as functional studies of candidate SNPs. Our approach is not intended to solve this problem (but see e.g. [48], [49] for reviews of methods for identifying causal variants after GWAS) and in our study the increases detection rate for parameters is accompanied by an expected increased false positive rate (Figure S1 and Figure S5 for the 200 SNPs case). However, as parameters as a rule are closer to mechanism than higher-level phenotypes, it should be noted that to do GWAS on parameters could become very instrumental for identifying candidate mechanisms and genes for follow up studies. We envision that ongoing efforts such as the RICORDO project [50] aimed at developing semantic interoperability for biomedical data and models will facilitate bioinformatic identification of candidate mechanisms and genes from cGP model sensitivities and GWAS results on parameter phenotypes.

We made deliberate use of the simplest possible genotype to parameter map in this study. A more complex map incorporating genetic dominance and various types of epistasis [51] would have diminished the SNP discovery rates and the explained variances of the parameters. However, this reduction in penetrance would apply equally well at higher phenotypic levels, and so would not affect our conclusions. We did not put any environmental variation on the parameters as we deemed this unnecessary in a context where the main focus was to compare the genetic signal strength at the parameter and cellular phenotype levels. However, in future studies this aspect needs to be taken into account in order to make quantitative assessments of how well we will be able to pick up genetic signals as function of environmental variation.

Our approach will remain useful in conjunction with future advances in statistical GWAS methodology, as it is applicable to any phenotypic variation that can be described by computational physiological modeling, irrespective of its position in the phenotypic hierarchy. Even in those cases where the parameters of a computational model are quite high-level phenotypes, our results suggest that one will be able to gain insights about the genotype to phenotype map that would otherwise be challenging to achieve.

There has been an enormous expansion in efforts to model complex biological systems the last decade, and steadily expanding model repositories such as http://www.cellml.org and http://biomodels.net facilitate exchange and reuse of such models. Illustratively, our study benefited from the reuse of a model available in CellML format. Future development of the cGP approach and systems genetics in general will benefit greatly from these standards and online resources as well as modeling efforts like the Virtual Physiological Human (http://www.vph-noe.eu).

Reflecting upon how to improve the current performance of large-scale GWA studies aiming to find the genetic determinants underlying complex diseases, Dermitzakis and Clark stated recently that “A major breakthrough will be to predict and interpret the effect of mutational and biochemical changes in human cells and understand how this signal is transmitted spatially (among tissues) and temporally (spanning development)” [52]. Our results suggest that combining GWAS methodology with a mature phenomics technology guided to fit the needs of computational physiology [53], may contribute substantially to making this vision come true.

## Supporting Information

Figure S1 **False positive rates of GWAS on parameters and cellular phenotypes (400 SNPs case).** Boxplots summarizing false positive rates (y axis) for 100 Monte Carlo simulations for (**A**) parameters and (**B**) cellular phenotypes. The false positive rate is defined as the proportion of the non-causative SNPs among those identified as significant by the GWAS.
(EPS)

Figure S2 **Percentage of causative SNPs detected by GWAS (200 SNPs case).** (**A**) The percentage of 200 causative SNPs (y axis) detected as significant SNPs by GWAS on genetically controlled model parameters (x axis). (**B**) The percentage of all 6800 causative SNPs (y axis) detected as significant SNPs by GWAS on cellular phenotypes (x axis). Each boxplot summarizes 100 Monte Carlo runs. See Methods for further descriptions of model parameters and phenotypes.
(EPS)

Figure S3 **Phenotypic variance explained by genotypic variation (200 SNPs case).** (**A**) Total explained variance for genetically controlled parameters (x axis) using detected causal SNPs as predictors. (**B**) Total explained variance for cellular phenotypes (x axis) using detected causal SNPs obtained from GWAS targeting these phenotypes. (**C**) Total explained variance for cellular phenotypes (x axis) using detected causal SNPs obtained from GWAS targeting all genetically controlled parameters. Each boxplot summarizes total explained variance by GWAS for 100 Monte Carlo runs. Explained variance was measured as R^2^ from test set prediction with a multiple regression model, see Methods for further descriptions.
(EPS)

Figure S4 **The close resemblance between GWAS results and linear sensitivity analysis (200 SNPs case).** (**A**) The number of causative SNPs for each parameter (y axis) detected by performing GWAS on high-level cellular phenotypes (x axis). The color intensity of each square describes the mean value of 100 Monte Carlo runs. (**B**) Sensitivities of the high-level phenotypes (x axis) of the 2500 individuals in the training set to variation in each parameter (y axis) quantified by univariate linear regression (see Methods). The color intensity of each square describes the mean R^2^ (coefficient of determination) value of 100 Monte Carlo runs.
(EPS)

Figure S5 **False positive rates of GWAS on parameters and cellular phenotypes (200 SNPs case).** Boxplots summarizing false positive rates (y axis) for 100 Monte Carlo simulations for (**A**) parameters and (**B**) cellular phenotypes. The false positive rate is defined as the proportion of the non-causative SNPs among those identified as significant by the GWAS.
(EPS)

Table S1 **Parameters with genetic variation.** This supplementary table contains data similar to that shown in Table 1, the only difference being that it is based on 200 causal SNPs per parameter instead of 400.
(PDF)

Table S2 **Attained cellular phenotype values.** This supplementary table contains data similar to that shown in Table 2, the only difference being that it is based on 200 causal SNPs per parameter instead of 400.
(PDF)

## References

[1] Maher B (2008). Personal genomes: The case of the missing heritability.. Nature.

[2] Manolio TA, Collins FS, Cox NJ, Goldstein DB, Hindorff LA (2009). Finding the missing heritability of complex diseases.. Nature.

[3] Makowsky R, Pajewski NM, Klimentidis YC, Vazquez AI, Duarte CW (2011). Beyond missing heritability: prediction of complex traits.. PLoS Genet.

[4] Lango Allen H, Estrada K, Lettre G, Berndt SI, Weedon MN (2010). Hundreds of variants clustered in genomic loci and biological pathways affect human height.. Nature.

[5] Park J-H, Wacholder S, Gail MH, Peters U, Jacobs KB (2010). Estimation of effect size distribution from genome-wide association studies and implications for future discoveries.. Nat Genet.

[6] Eichler EE, Flint J, Gibson G, Kong A, Leal SM (2010). Missing heritability and strategies for finding the underlying causes of complex disease.. Nat Rev Genet.

[7] Yang J, Manolio TA, Pasquale LR, Boerwinkle E, Caporaso N (2011). Genome partitioning of genetic variation for complex traits using common SNPs.. Nat Genet.

[8] Zeggini E, Scott LJ, Saxena R, Voight BF, Marchini JL (2008). Meta-analysis of genome-wide association data and large-scale replication identifies additional susceptibility loci for type 2 diabetes.. Nat Genet.

[9] Cho YS, Chen C-H, Hu C, Long J, Hee Ong RT (2011). Meta-analysis of genome-wide association studies identifies eight new loci for type 2 diabetes in east Asians.. Nat Genet.

[10] Kato N, Takeuchi F, Tabara Y, Kelly TN, Go MJ (2011). Meta-analysis of genome-wide association studies identifies common variants associated with blood pressure variation in east Asians.. Nat Genet.

[11] Burns J, Waddington CH (1970). The synthetic problem and the genotype-phenotype relation in cellular metabolism.. Towards a Theoretical Biology. 3. Drafts. An I.U.B.S. Symposium.

[12] Frank SA (1999). Population and Quantitative Genetics of Regulatory Networks.. J Theor Biol.

[13] Omholt SW, Plahte E, Oyehaug L, Xiang K (2000). Gene regulatory networks generating the phenomena of additivity, dominance and epistasis.. Genetics.

[14] Gilchrist MA, Nijhout HF (2001). Nonlinear developmental processes as sources of dominance.. Genetics.

[15] Ma C-X, Casella G, Wu R (2002). Functional mapping of quantitative trait loci underlying the character process: a theoretical framework.. Genetics.

[16] Peccoud J, Velden KV, Podlich D, Winkler C, Arthur L (2004). The selective values of alleles in a molecular network model are context dependent.. Genetics.

[17] Welch SM, Dong Z, Roe JL, Das S (2005). Flowering time control: gene network modelling and the link to quantitative genetics: Modelling complex traits for plant improvement.. Aust J Agric Res.

[18] Gjuvsland AB, Hayes BJ, Omholt SW, Carlborg O (2007). Statistical epistasis is a generic feature of gene regulatory networks.. Genetics.

[19] Rajasingh H, Gjuvsland AB, Våge DI, Omholt SW (2008). When parameters in dynamic models become phenotypes: a case study on flesh pigmentation in the chinook salmon (*Oncorhynchus tshawytscha*).. Genetics.

[20] Gertz J, Gerke JP, Cohen BA (2010). Epistasis in a quantitative trait captured by a molecular model of transcription factor interactions.. Theor Popul Biol.

[21] Salazar-Ciudad I, Jernvall J (2010). A computational model of teeth and the developmental origins of morphological variation.. Nature.

[22] Pumir A, Shraiman B (2011). Epistasis in a Model of Molecular Signal Transduction.. PLoS Comput Biol.

[23] Fu G, Wang Z, Li J, Wu R (2011). A mathematical framework for functional mapping of complex phenotypes using delay differential equations.. J Theor Biol.

[24] Vik JO, Gjuvsland AB, Li L, Tøndel K, Niederer S (2011). Genotype-phenotype map characteristics of an in silico heart cell.. Front Physio.

[25] Gjuvsland AB, Plahte E, Omholt SW (2007). Threshold-dominated regulation hides genetic variation in gene expression networks.. BMC Syst Biol.

[26] Gieger C, Geistlinger L, Altmaier E, Hrabé de Angelis M, Kronenberg F (2008). Genetics meets metabolomics: a genome-wide association study of metabolite profiles in human serum.. PLoS Genet.

[27] Illig T, Gieger C, Zhai G, Römisch-Margl W, Wang-Sattler R (2009). A genome-wide perspective of genetic variation in human metabolism.. Nat Genet.

[28] Baranzini SE, Galwey NW, Wang J, Khankhanian P, Lindberg R (2009). Pathway and network-based analysis of genome-wide association studies in multiple sclerosis.. Hum Mol Genet.

[29] Cookson W, Liang L, Abecasis G, Moffatt M, Lathrop M (2009). Mapping complex disease traits with global gene expression.. Nat Rev Genet.

[30] Ala-Korpela M, Kangas AJ, Inouye M (2011). Genome-wide association studies and systems biology: together at last.. Trends Genet.

[31] Das K, Li J, Wang Z, Tong C, Fu G (2011). A dynamic model for genome-wide association studies.. Hum Genet.

[32] Noble D (2002). Modeling the Heart–from Genes to Cells to the Whole Organ.. Science.

[33] Smith NP, Mulquiney PJ, Nash MP, Bradley CP, Nickerson DP (2002). Mathematical modelling of the heart: cell to organ.. Chaos, Solitons & Fractals.

[34] Smith NP, Nickerson DP, Crampin EJ, Hunter PJ (2004). Multiscale computational modelling of the heart.. ANU.

[35] Hunter PJ, Borg TK (2003). Innovation: Integration from proteins to organs: the Physiome Project.. Nat Rev Mol Cell Biol.

[36] Nickerson D, Nash M, Nielsen P, Smith N, Hunter P (2006). Computational multiscale modeling in the IUPS Physiome Project: Modeling cardiac electromechanics.. IBM J Res & Dev.

[37] Smith N, de Vecchi A, McCormick M, Nordsletten D, Camara O (2011). euHeart: personalized and integrated cardiac care using patient-specific cardiovascular modelling.. Interface Focus.

[38] Gibbs RA, Belmont JW, Hardenbol P, Willis TD, Yu F (2003). The International HapMap Project.. Nature.

[39] Altshuler DM, Gibbs RA, Peltonen L, Altshuler DM, Gibbs RA (2010). Integrating common and rare genetic variation in diverse human populations.. Nature.

[40] Li L, Niederer SA, Idigo W, Zhang YH, Swietach P (2010). A mathematical model of the murine ventricular myocyte: a data-driven biophysically based approach applied to mice overexpressing the canine NCX isoform.. Am J Physiol Heart Circ Physiol.

[41] Bondarenko VE, Szigeti GP, Bett GCL, Kim S-J, Rasmusson RL (2004). Computer model of action potential of mouse ventricular myocytes.. Am J Physiol Heart Circ Physiol.

[42] Peng B, Amos CI (2010). Forward-time simulation of realistic samples for genome-wide association studies.. BMC Bioinformatics.

[43] Lloyd CM, Halstead MDB, Nielsen PF (2004). CellML: its future, present and past.. Prog Biophys Mol Biol.

[44] Cohen S, Hindmarsh C (1996). CVODE, a stiff/nonstiff ODE solver in C.. Computers in physics.

[45] Purcell S, Neale B, Todd-Brown K, Thomas L, Ferreira MAR (2007). PLINK: a tool set for whole-genome association and population-based linkage analyses.. Am J Hum Genet.

[46] Aulchenko YS, Struchalin MV, Belonogova NM, Axenovich TI, Weedon MN (2009). Predicting human height by Victorian and genomic methods.. Eur J Hum Genet.

[47] Saltelli A, Ratto M, Andres T, Campolongo F, Cariboni J (2008). Global sensitivity analysis: the primer.

[48] Cantor RM, Lange K, Sinsheimer JS (2010). Prioritizing GWAS results: A review of statistical methods and recommendations for their application.. Am J Hum Genet.

[49] Ioannidis JPA, Thomas G, Daly MJ (2009). Validating, augmenting and refining genome-wide association signals.. Nat Rev Genet.

[50] de Bono B, Hoehndorf R, Wimalaratne S, Gkoutos G, Grenon P (2011). The RICORDO approach to semantic interoperability for biomedical data and models: strategy, standards and solutions.. BMC Res Notes.

[51] Phillips PC (2008). Epistasis — the essential role of gene interactions in the structure and evolution of genetic systems.. Nat Rev Genet.

[52] Dermitzakis ET, Clark AG (2009). Genetics. Life after GWA studies.. Science.

[53] Houle D, Govindaraju DR, Omholt S (2010). Phenomics: the next challenge.. Nat Rev Genet.

