# Supplementary material for: Parameters in Dynamic Models of Complex Traits are Containers of Missing Heritability
Source: PLoS Comput Biol. 2012 Apr 5;8(4):e1002459. doi: 10.1371/journal.pcbi.1002459 (PMC3320574; doi:10.1371/journal.pcbi.1002459)
Supplement: Table S1 — Parameters with genetic variation. This supplementary table contains data similar to that shown in Table 1, the only difference being that it is based on 200 causal SNPs per parameter instead of 400. (PDF) [file pcbi.1002459.s006.pdf]

Table S1. Parameters with genetic variation (200 SNPs case). Listing of the 34 parameters where genetic variation was introduced. The descriptions, units and baseline values are taken from the original publication [40]. The minimum and maximum values were obtained from the Monte Carlo simulations.

Parameter	Description	Unit	Baseline value	Min	Max
**Ka+**	The PC1 – PO1 rate constant of the Ryanodine receptor	$\mu M^{-4}/ms$	6.08e-3	4.16e-3	8.31e-3
**Ka-**	The PO1 – PC1 rate constant of the Ryanodine receptor	ms^{-1}	7.133-2	4.52e-2	9.59e-2
**Kb+**	The PO1 – PO2 rate constant of the Ryanodine receptor	$\mu M^{-3}/ms$	4.05e-3	2.72e-3	5.45e-3
**Kb-**	The PO2 – PO1 rate constant of the Ryanodine receptor	ms^{-1}	9.65e-1	6.59e-1	1.27
**Kc+**	The PO1 – PC2 rate constant of the Ryanodine receptor	ms^{-1}	9.00e-3	5.81e-3	1.17e-2
**Kc-**	The PC2 – PO1 rate constant of the Ryanodine receptor	ms^{-1}	8.00e-4	5.50e-4	1.12e-3
**m**	The Ca^{2+} cooperativity parameter of PO1 – PO2 of the Ryanodine receptor	-	3.0	2.03	4.02
**n**	The Ca^{2+} cooperativity parameter of PC1 – PO1 of the Ryanodine receptor	-	4.0	2.68	5.44
**P_CaL**	The permeability of the L-type Ca^{2+} channel	ms^{-1}	2.5	1.66	3.33
**t_L**	The time constant of the switch between open and close states of the L-type Ca^{2+} channel	ms^{-1}	1.5	9.21e-1	2.00
**tau_L**	The Inactivation time constant of the L-type Ca^{2+} channel	ms^{-1}	1.15e3	7.51e2	1.54e3
**phi_L**	The proportion of closed states in open mode of the L-type Ca^{2+} channel	-	1.80	1.22	2.42
**Kup**	The SERCA affinity to Ca^{2+}	µM	4.12e-1	2.80e-1	5.43e-1
**V1**	The leak constant of the Network Sarcoplasmic Reticulum	ms^{-1}	4.5	3.04	5.92
**KCSQN**	The Calsequestrin affinity to Ca^{2+}	µM	6.30e2	4.08e2	8.54e2
**K_Co**	The affinities of the Na^+/Ca^{2+} exchanger to extracellular Ca^{2+}	µM	1.4e3	9.53e2	1.86e3
**K_Ci**	The affinities of the Na^+/Ca^{2+} exchanger to intracellular Ca^{2+}	µM	3.6	2.47	4.85
**K_No**	The affinities of the Na^+/Ca^{2+} exchanger to extracellular Na^+	µM	8.80e4	5.92e04	1.15e5
**K_Ni**	The affinities of the Na^+/Ca^{2+} exchanger to intracellular Na^+	µM	1.2e4	8.00e3	1.60e4
**KNai**	The affinity of the Na^+/K^+ pump to intracellular Na^+	µM	1.66e4	1.13e4	2.20e4
**KKo**	The affinity of the Na^+/K^+ pump to extracellular K^+	µM	1.5e3	1.03e3	1.98e3
**KpCa**	The affinity of the Ca^{2+} pump to intracellular Ca^{2+}	µM	2.89e-1	1.94e-1	4.03e-1
**Vmax**	The maximal exchange rate of Na^+/Ca^{2+} exchanger	pA/pF	3.94	2.73	5.22
**Imax**	The maximal current of the Na^+/K^+ pump	pA/pF	2.49	1.56	3.33
**GK1**	The maximal conductance of the time-dependent K^+ channel	ms/µF	3.5e-1	2.32e-1	4.66e-1
**GKr**	The maximal conductance of the rapid delayed rectifier K^+ channel	ms/µF	1.65e-2	1.12e-2	2.23e-2
**GKur**	The maximal conductance of the ultrarapidly activating delayed rectifier K^+ channel	ms/µF	2.50e-1	1.68e-1	3.33e-1
**KCl**	The half saturation constant of the Ca^{2+} activated Cl^- channel	µM	10.0	6.30	1.32e1

**GNa**	The maximal conductance of the Na^+ channel	ms/µF	1.60e1	1.03e1	2.17e1
**GKtof**	The maximal conductance of the rapidly recovering transient outward K^+ channel	ms/µF	5.35e-1	3.54e-1	7.16e-1
**GClCa**	The maximum conductance of the Ca^{2+} activated Cl^- channel	ms/µF	1.00e1	6.04	1.33e1
**on_rate**	The autophosphorylation rate of Calmodulin	ms^{-1}	5.0e-2	3.07e-2	6.50e-2
**off_rate**	The dephosphorylation rate of the Calmodulin	ms^{-1}	2.0e-4	1.31e-4	2.67e-4
**IpCm**	The maximal current of the Ca^{2+} pump	pA/pF	9.55e-2	6.14e-2	1.28e-1
