# Supplementary material for: Parameters in Dynamic Models of Complex Traits are Containers of Missing Heritability
Source: PLoS Comput Biol. 2012 Apr 5;8(4):e1002459. doi: 10.1371/journal.pcbi.1002459 (PMC3320574; doi:10.1371/journal.pcbi.1002459)
Supplement: Table S2 — Attained cellular phenotype values. This supplementary table contains data similar to that shown in Table 2, the only difference being that it is based on 200 causal SNPs per parameter instead of 400. (PDF) [file pcbi.1002459.s007.pdf]

Table S2. Attained cellular phenotype values (200 SNPs case).
The phenotypic values resulting from use of the baseline parameter values (see Table S1) are listed together with the minimum and maximum values achieved in the Monte Carlo simulations.

Phenotypes	Unit	Baseline value	Min	Max
**apd25**	ms	4.34	4.10	4.58
**apd50**	ms	5.89	5.34	6.41
**apd75**	ms	1.11e1	9.32	1.29e1
**apd90**	ms	1.95e1	1.62e1	2.30e1
**apamp**	mV	1.18e2	1.14e2	1.22e2
**apbase**	mV	-8.0e1	-8.07e1	-7.93e1
**appeak**	mV	3.82e1	3.42e1	4.20e1
**apttp**	ms	3.20	3.04	3.34
**ctd25**	ms	6.19e1	4.72e1	7.78e1
**ctd50**	ms	1.05e2	8.0e1	1.33e2
**ctd75**	ms	1.79e2	1.39e1	2.23e2
**ctd90**	ms	2.55e2	2.20e2	2.77e2
**ctamp**	µM	1.40e-1	5.05-2	2.74e-1
**ctbase**	µM	8.14e-2	6.10e-2	1.06e-1
**ctpeak**	µM	2.21e-1	1.15e-1	3.68e-1
**ctttp**	ms	2.40e1	1.92e1	2.94e2
